# Adaptation in Practice: How Managers of Nature Conservation Areas in Eastern England are Responding to Climate Change

**DOI:** 10.1007/s00267-014-0254-6

**Published:** 2014-03-20

**Authors:** Nicholas A. Macgregor, Nikki van Dijk

**Affiliations:** 1 Natural England, Nobel House, 17 Smith Square, London, SW1P 3JR UK; 2 Atkins, Western House (Block B), Peterborough Business Park, Lynch Wood, Peterborough, PE2 6FZ UK

**Keywords:** Climate change adaptation, Nature conservation, Adaptive management, Nature reserves, Protected areas, Resilience, Transformative adaptation

## Abstract

Although good general principles for climate change adaptation in conservation have been developed, it is proving a challenge to translate them into more detailed recommendations for action. To improve our understanding of what adaptation might involve in practice, we investigated how the managers of conservation areas in eastern England are considering climate change. We used a written questionnaire and semi-structured interviews to collect information from managers of a range of different conservation areas. Topics investigated include the impacts of climate change perceived to be of the greatest importance; adaptation goals being set; management actions being carried out to achieve these goals; sources of information used; and perceived barriers to taking action. We identified major themes and issues that were apparent across the sites studied. Specifically, we found ways in which adaptation had been informed by past experience; different strategies relating to whether to accept or resist change; approaches for coping with more variable conditions; ways of taking a large-scale approach and managing sites as networks; some practical examples of aspects of adaptive management; and examples of the role that other sectors can play in both constraining and increasing a conservation area’s capacity to adapt. We discuss the relevance of these findings to the growing discussion in conservation about identifying adaptation pathways for different conservation areas and a potential progression from a focus on resilience and incremental change to embracing “transformation.” Though adaptation will be place-specific, we believe these findings provide useful lessons for future action in both England and other countries.

**Electronic supplementary material:**

The online version of this article (doi:10.1007/s00267-014-0254-6) contains supplementary material, which is available to authorized users.

## Introduction

The effects of climate change on species and ecosystems in England and other countries are already being detected (e.g., Hickling et al. 2006; Rosenzweig et al. 2008; Walther 2010; Morecroft and Speakman 2013). These effects can be expected to grow more serious over time as the climate continues to change. There is growing recognition that adaptation to climate change requires serious consideration in conservation policy and practice (Defra 2011a; European Commission 2012).

A range of good adaptation principles and recommendations have been developed to help in guiding nature conservation in a changing climate (Hopkins et al. 2007; Huntley 2007; Smithers et al. 2008; Heller and Zavaleta 2009; Lawler 2009; Mawdsley et al. 2009). These ideas appear now to be reasonably well established in conservation thinking. However, many of the published recommendations are quite general, a point that has been highlighted to us in feedback from managers of conservation areas. There is a need to go beyond general principles to develop recommendations for adaptation that are sufficiently specific to different areas, circumstances, landscape types, ecosystems, and ecological assemblages, and thus more practically useful for conservation practitioners on the ground.

At the other end of the spectrum, there are a small number of useful site-specific case studies (e.g., Flux 2009), but more needs to be done to draw out general conclusions and themes that can be applied to other places. In other words, one challenge is to bridge the gap between insufficiently specific principles, on the one hand, and very specific local case studies on the other—something that has been noted in other fields of adaptation (Howden et al. 2010). (See Hansen et al. 2010 for an example of a paper that does attempt to make this link between principles and case studies.)

To contribute to addressing this issue, and to explore what practical adaptation might involve in different places, there is potential to learn from the ways in which the managers of conservation areas might already be starting to consider adaptation to climate change. In many cases, the approaches they are taking are likely to build on past experiences of coping with natural fluctuations in the environment, and could provide a foundation for considering and evaluating options for adapting to future conditions.

We have attempted to do this by investigating how adaptation is being approached by the managers of conservation sites in England. Our primary aim is to understand better the different ways in which nature conservation can adapt in practice and the issues involved. An underlying question is under which circumstances adaptation will require radically new actions rather than slight modifications to the existing conservation practice. When is “incremental” adaptation sufficient, and when is “transformative” adaptation (Kates et al. 2012; O’Brien 2012; Palutikof et al. 2013) needed?

The focus of our study is East Anglia, in eastern England. East Anglia is a low-lying area with a soft coastline facing potentially high rates of sea level rise and coastal flooding (Holman et al. 2002; Murphy et al. 2009; Brown et al. 2012). It has a low level of rainfall, and it includes some of the driest places in England (Met Office 2013). East Anglia contains some of England’s most important lowland freshwater wetlands in The Fens and in the Norfolk Broads, and other coastal areas in Norfolk and Suffolk; valuable inter-tidal habitats on the Norfolk, Suffolk, and Essex coasts; and important dry heathland ecosystems in the Breckland area. It supports a diverse range of species, including many that are a priority for conservation or are found almost nowhere else in the United Kingdom (Dolman et al. 2010; Panter et al. 2011; Mossman et al. 2012).

East Anglia experienced increases in summer temperature above the national average during the period 1961–2006 (Jenkins et al. 2008). Previous studies (e.g., Natural England 2009) have suggested that the impacts of climate change on the natural environment in this part of England could be among the most serious in the country, and some conservation areas in East Anglia have experienced extreme weather events (including river flooding, coastal flooding, and drought) in recent years. Thus, it provided a good study area to explore the practical issues and decisions involved in adaptation, as we might expect conservation managers here to have considered environmental change and variability to a greater extent than their counterparts in other areas of the country.

We used a questionnaire and interviews to collect information about the climate impacts that are of the greatest concern to site managers, the adaptation goals they are formulating in response, how these goals influence action on the ground, the sources of information that are of most use, and the perceived barriers to taking action.

## Methods

**Fig. 1:**
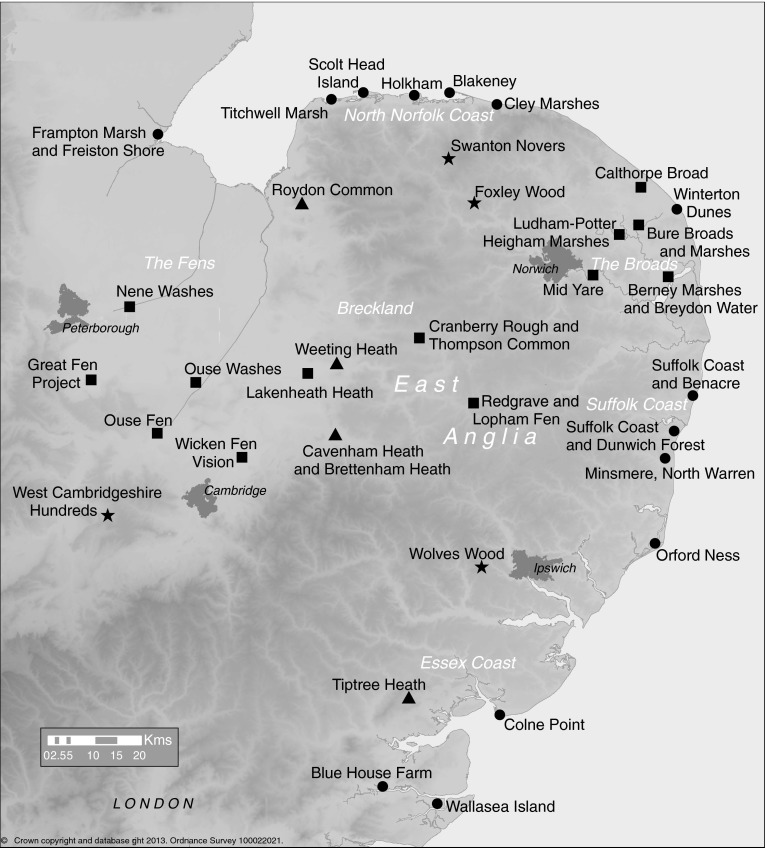
Map of East Anglia, showing the approximate location of the 35 sites that were surveyed in this study. Sites are categorized by location and the broad ecosystem/land cover type that is of greatest geographic extent or conservation interest at each site: *circles* coastal; *squares* (inland) freshwater wetland; *stars* woodland; *triangles* heathland. Some sites contained multiple geographic locations in close proximity; in these cases, the map shows only one of these locations. Multiple locations are indicated in some site labels but had to be omitted in some cases, because of limited space. The map also shows the general location of six areas within East Anglia that are mentioned in the text of the paper: the Fens, Breckland, North Norfolk coast, the Broads, Suffolk coast, and Essex coast

The study centered on the East Anglia region in eastern England (Fig. 1). We invited six major conservation organizations to take part in the survey: Natural England; the Royal Society for the Protection of Birds (RSPB); the National Trust; the Norfolk Wildlife Trust; the Suffolk Wildlife Trust; the Essex Wildlife Trust; and the Wildlife Trust for Bedfordshire, Cambridgeshire, and Northamptonshire. The first of these is a government conservation agency; the others are independent conservation organizations. Between them, they are responsible for managing the majority of nature reserves and other conservation areas in this part of England.

These organizations were asked to suggest conservation sites in East Anglia for inclusion in the study, and the names of individual staff to contact. Invitations to participate in the survey were then sent to individual staff members. In most cases, they were nature reserve managers, in a few cases regional conservation officers with responsibility for advising staff in a number of reserves in the same area.

We conducted a survey, in two parts. The first part consisted of a written questionnaire, designed to collect categorical information through the use of yes/no or multiple choice answers, with space for respondents also to provide notes. The questionnaire covered six broad topics: background information about the conservation site and its overall conservation objectives; climate impacts of concern; adaptation goals; management actions; information sources used; and barriers to action (see supplementary material).

Respondents were asked to complete a separate questionnaire form for each conservation site, unless they had a cluster of similar conservation areas that were in close proximity and facing similar adaptation issues (in which case the group of areas could be considered a single “site” for the purposes of the survey). Questionnaires were completed by conservation staff between January 27, 2012 and 13 March 13, 2012.

The second part of the survey was an interview, conducted over the phone, with each person who had completed the survey (if they had indicated their willingness to be contacted). Interviews were conducted between February 13, 2012 and March 15, 2012. The interviews were led by two interviewers, and followed a semistructured format. The questions and answers on the completed questionnaire form were used as a basis, but the interview was allowed to flow as a conversation around those topics. More time was spent on whichever topics emerged as being the most interesting for the particular site being discussed, and, as far as possible, interviewees were invited simply to expand on the written answers they had provided (Valentine 1997). As well as providing an opportunity for both interviewers and interviewees to review the questionnaire form and clarify original answers or fill in gaps, the interview format proved an effective way of elucidating key points, providing additional information to supplement the information in the questionnaire, and making links between issues. Both interviewers took detailed notes during interviews, which were transcribed and combined as soon as possible afterward.

The categorical information collected from the written questionnaire was used to generate simple summary data. From the key phrases and information in the interview notes, examples were identified to supplement the questionnaire results. Major themes that were apparent across a large number of sites in the survey were identified, as were examples of sites that illustrated a particularly striking or interesting aspect of each theme.

Questionnaires were returned for 35 conservation sites (of which some were groups of nearby reserves), by 28 individual respondents (i.e., some respondents provided information for multiple sites). This represents a response rate of ~60 % from among the individual conservation staff we contacted. Interviews were held with 26 people (the other two being too busy with work commitments to take part).

The sites for which information was collected are spread widely across East Anglia and are a fairly representative sample of the major ecosystem/land cover types found in this part of England. Many sites contain multiple vegetation or ecosystem types. However, based on their locations and on the ecosystem that occupies the greatest area and/or is of greatest conservation concern in each site, they can, in very broad terms, be categorized into four types: coastal sites (sites that are on the coastline or very close to it; many of these contain important freshwater wetlands in close proximity to the sea); inland freshwater wetland sites; (inland) heathland sites; and (inland) woodland sites (Fig. 1). Sites ranged in size from less than 50 ha to more than 3,000 ha (and some even bigger proposed restoration areas); and ranged in age from old sites established approximately a century ago to new restoration projects established within the last decade.

Many of the sites were at least partly covered by legal conservation designations: more than 90 % contained Sites of Special Scientific Interest, 60 % contained National Nature Reserves, and more than 60 % contained Special Areas of Conservation, Special Protection Areas, or Ramsar sites. However, some of the sites also contained areas with no formal conservation designations; this was particularly the case at newly-created sites or in ecological restoration areas adjacent to older reserves. Many of the sites on or near the coast were also in areas designated as National Park, Heritage Coast or Area of Outstanding Natural Beauty. (For definitions of all conservation designations mentioned above, see Natural England 2008.)

Results presented in this paper include both summaries of categorical data from the questionnaire and more detailed qualitative information from the interviews. Although some respondents provided information for multiple sites under their management and therefore data for these sites might not be independent, for most questions, respondents gave different information specific to each site. Therefore, most categorical data are presented on a site-by-site basis. The exception is the data on information sources, for which at least one respondent provided a general response about information he had found useful across all his sites. For this topic, therefore, results are presented by “site manager” instead of by “site.” For some of the topics we present information both for all sites and for different types of sites (coastal sites; inland wetland sites; woodland; and heathland sites combined). The focus of the study was on gaining qualitative insights, and the sample sizes and nature of some of the data limit the value of formal statistical tests, but we note where there were striking differences in survey responses among the different site types.

The locations and types of sites surveyed are shown in Fig. 1, but when examples are given in the text individual sites are generally not identified by name, except in a few cases in which previously-published information about a particular site is cited. Some sites are also named in the photo captions with permission from site managers. The terms “respondent” and “site manager” are used interchangeably to refer to people who took part in the survey, with “interviewee” also used when talking specifically about interviews.

## Results

### Vulnerability Assessments and Climate Impacts of Concern

Managers of 57 % of sites had done, or were aware of, a “simple” vulnerability assessment for the site (either a basic assessment for the site itself based on general ecological principles, or drawing information from external assessments done for nearby sites or for the overall area in which the site was located). In another 17 % of sites, a “detailed” site-specific assessment had been undertaken. In some cases, the vulnerability assessment had been done in coordination with other conservation sites nearby. When a vulnerability assessment had not been done, information about climate impacts of concern provided by survey respondents presumably drew on informal assessments or personal opinion.

**Fig. 2:**
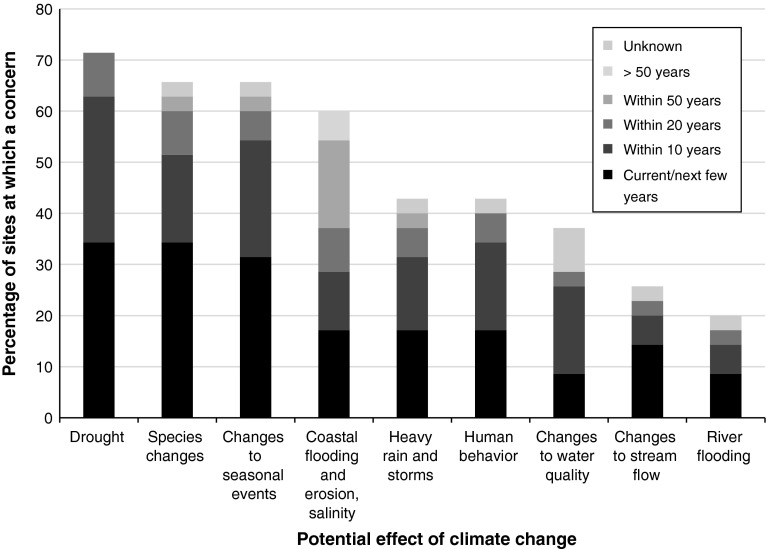
Percentage of sites at which different climate impacts were cited as being a concern for conservation management either now or in the future (with respondents choosing as many options as relevant from a set of predetermined categories). The time periods shown indicate the point in time at which respondents thought the impact in question would become an issue at the site. Data are shown for all 35 sites

The effects of climate change most frequently identified by site managers as presenting issues for management of their sites were drought, species changes, changes to seasonal events, and coastal flooding, and associated impacts. (Of these, interviews revealed drought and coastal change to be the ones that managers were most concerned about; the latter was a concern not only at many coastal sites but also at some further inland that could potentially be affected by storm surges.) However, each category of impact listed in the questionnaire was selected by respondents in at least five sites (Fig. 2).

Climate change was indicated to be a concern “now or in the next few years” at 24 sites (69 % of the total), and at one other site an urgent problem related to coastal erosion had already been dealt with through coastal realignment before our survey took place. Recent or current extreme events, and examples of their conservation implications, reported to us included:

- Unpredictable rainfall in recent years with extremes of wet and dry conditions. This had led to, for example, delays in bird fledging in very wet springs, and easier access for nest predators during dry periods.
- Drought. At the time of the survey, East Anglia was experiencing a severe drought, and there was evidence of the effects on habitats and species. For example, natterjack toads (*Epidalea calamita)* were not expected to breed in 2012 at a number of sites.
- Milder winters/warmer springs and earlier vegetation growth. At one site, this was reported to have led to grass growth before birds were ready to nest, and a consequent decline in the breeding success of lapwings (*Vanellus vanellus)*; at another site it had led to more rapid spread of invasive holly.
- Past coastal or tidal flooding, causing saline water to infiltrate freshwater ecosystems, from which in some cases it had been hard to remove.
- Increased seasonal river flooding, submerging riparian grassland used by breeding wading birds.

**Fig. 3:**
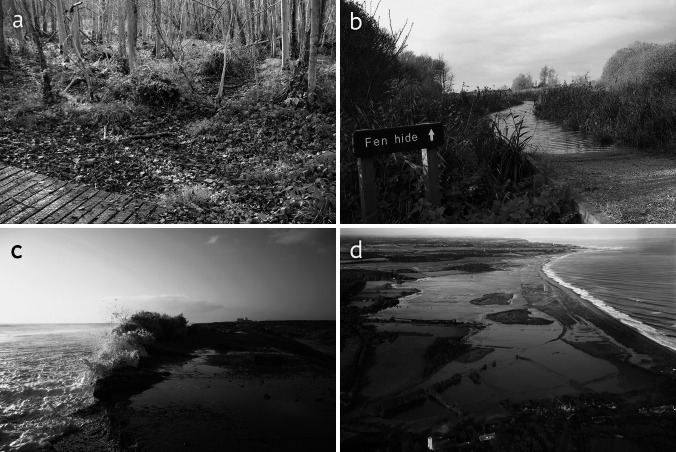
Some of the recent environmental pressures faced at sites surveyed in the study. **a** A dry stream in the valley alder wood in Foxley Wood, Norfolk, thought to be the result of drought and/or local water abstraction (John Milton, Norfolk Wildlife Trust); **b** saline flooding of reedbed at Strumpshaw Fen in the Mid Yare reserve, as a result of water backing up the River Yare following an exceptional surge tide event (RSPB); **c** a storm surge breaching the coastal defenses in front of Minsmere Nature Reserve in Suffolk (Robin Harvey, RSPB); **d** major coastal flooding of freshwater wetland at the Suffolk Coast National Nature Reserve (www.mikepage.com)

Some of these are illustrated in Fig. 3. While it is important to note that many of these individual current or recent events could not be conclusively attributed to climate change, they are similar to the impacts that site managers said they expected from climate change; in many cases, climate could be an influencing factor, or become one.

At some sites, climate change was thought likely to exacerbate some of the existing problems caused by human activities outside the site. For example, climate change-induced drought might worsen the existing pressures on some sites as a result of increased abstraction of water, or cause low water levels that would exacerbate water quality problems caused by nearby livestock farming.

### Extent to Which Adaptation was Part of Conservation Planning and Management

At 17 sites (49 % of the total), adaptation was reported to be a major part of planning and management. Of those, at four sites it had been part of site management since the site was established (in all cases at least 6 years previously). At an additional 16 sites (46 % of the total), adaptation was reported to be a consideration in planning and management, but a relatively minor factor. At one of these sites (an ecological restoration area established more than 10 years ago), adaptation had been part of management since the beginning. At the 33 sites at which adaptation was reported to be either a minor or major consideration, sites varied in how long adaptation had been a consideration in management and planning, with 3–5 years the modal response (Table 1).

**Table 1:** The length of time that adaptation had been considered in planning and management at different sites

Length of time that adaptation had been considered	Number of sites
Adaptation not currently being considered	2
1–2 years	2
3–5 years	14
6–10 years	7
More than 10 years	8
Unknown	2

Data are shown for all 35 sites

### Adaptation Goals

**Fig. 4:**
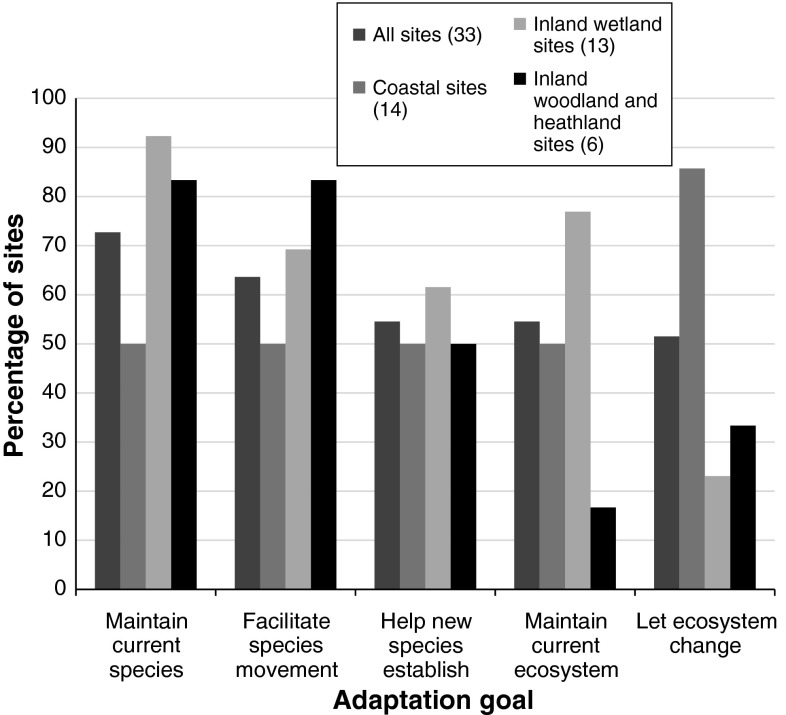
The percentage of sites that had particular adaptation goals. Data are shown—from *left to right*—for all 33 sites at which adaptation was being considered, coastal sites, inland wetland sites, and woodland/heathland sites. Goals were selected by survey respondents from a predetermined list, with respondents being able to select more than one for each site

The questionnaire asked respondents to select, from a list, adaptation goals that applied at their site. Of the options given, maintaining current species was the most commonly selected goal (73 % of the sites at which adaptation was being considered). The other four options (facilitating species movement, enabling new species to become established, maintaining the current ecosystem and letting the ecosystem change) were also selected for a large number of sites (Fig. 4). When responses are compared across the different types of site, the most noticeable difference is between coastal and inland wetland sites in the goals relating to maintaining or changing ecosystems; accepting or facilitating change was a much more common goal at coastal sites (86 %) than at inland wetland sites (23 %).

#### Sites Aiming to Maintain the Current Ecosystem

The goals adopted in some cases, were clearly linked to the level of certainty about future changes. At sites where managers had reasonable certainty that events were far off in time (e.g., sea defenses with a lifetime of 50 or more years, security over future water supplies, or confidence about the length of time it might take saline water to move inland), we found that the approach was generally to try to maintain current conditions. This was particularly the case: for sites that had a long history of being managed in a certain way; for sites that had a valued set of species that would be disturbed or lost if over-hasty changes were made; and where there would be value in maintaining the site in the short-medium term to buy time for establishment of new reserves and populations elsewhere. A number of examples of this strategy were seen at well-established freshwater sites near the coast but protected by strong sea walls. This was not always a passive continuation of past management. At some sites, significant engineering work had been undertaken to protect habitats from urgent threats and give confidence that further changes would not be needed for a long time.

We found a second group of sites at which there seemed to be an approach of maintaining the current ecosystems and species, but for quite different reasons. Here, expected changes appeared to be quite subtle (particularly the case in drier inland sites), or there was uncertainty over which of two or more opposing potential changes would prevail (for example, projected drier conditions reducing grass growth but warmer springs increasing it). The resulting management approach seemed to be one of watching and waiting, while doing everything possible to increase resilience to a wide range of possible future changes, and, in some cases, increasing knowledge about possible management.

#### Sites Allowing or Facilitating Ecosystem Change

By contrast with some of the examples mentioned above, at sites at which major change seemed likely and unavoidable in the short-medium term, a more flexible approach that accommodated change was apparent. At one site, established relatively recently, it was expected that the freshwater part of the site could be protected for only another 20 years. This recognition that freshwater habitats were unlikely to be sustainable in the long term was built into the design and management of the site. Wet grassland had been established in preference to more cost- and labor-intensive freshwater systems such as reedbed. As sea level rises, management was expected to change and the grazing marsh would be allowed to revert to salt marsh. (This contrasted with a nearby site managed by the same organization, where an analysis of the potential impacts of tidal flooding had concluded that the sea defenses had a lifetime of 50–100 years; and as a result it had been decided to develop and maintain reedbed (a slower and more expensive habitat to create), on the basis that it would be protected from sea level rise for long enough to justify its creation.)

In at least two cases, acceptance of change was based on an intentionally non-interventionist approach to conservation, driven by the view that natural coastal processes are dynamic and that change is a natural part of the functioning of the sites. At one site, one of the main conservation objectives was to research natural coastal functioning and the effect of sea level rise on saltmarsh. Therefore, there was virtually no management done and coastal processes were allowed to operate unhindered, with monitoring undertaken of consequent changes.

Acceptance of change included accommodating temporary changes and fluctuations as well as permanent change. At two sites in particular (one on the Suffolk coast, the other in the Norfolk Broads), there was increasing acceptance of variations in natural conditions from year to year and acceptance that some years would be better than others for different species. As a result, the targets for water levels and for sizes of populations of priority species were being set for periods of multiple years, rather than requiring a particular level or number to be met every year.

An even more flexible approach was apparent in two of the sites involving large-scale restoration. A low-intervention approach was being taken in the newly restored areas, with few or no targets for species or assemblages. As far as possible, natural processes were allowed to operate to reestablish natural habitat on former arable land, an approach that implicitly accepted natural variability.

Several interviewees indicated that they thought that change could bring some opportunities, such as new species that would have conservation value in themselves and for their contribution to the ecosystem. In some cases, direct impacts of climate change were thought potentially to benefit ecosystems, although there was often uncertainty about the direction of change and the opportunities climate change might bring. Others noted the opportunity to expand some habitat areas and reestablish natural processes if current interest features that would be likely to be lost could first be restored, enlarged or created elsewhere. For example, brackish wetlands and saline lagoons could expand at some coastal sites, which would not necessarily be a bad outcome if the freshwater systems they replaced could be created in other places. It was also pointed out that as the first sites are affected by particular pressures, we could learn more about how natural ecosystems change from one state to another and how individual species respond.

Newly arriving species (but not invasive non-native species) were being accommodated in many cases, although few sites reported actively managing for or encouraging new arrivals. In most cases, reserve managers appeared confident that by maintaining conditions for current species, habitat would be provided for many of the likely new arrivals (for example purple heron (*Ardea* *purpurea)*, little bittern (*Ixobrychus minutes)*, Mediterranean gull (*Larus melanocephalus)* and little egret (*Egretta garzetta)*). The manager of one woodland site told us that future conditions were being taken into account when considering possible re-introduction of butterfly species.

#### Sites with Multiple Adaptation Goals

Respondents were able to select more than one goal if relevant to the site in question. In several cases, multiple goals that indicate a balance between conservation of current biodiversity and accommodation of change were reported. For example, both “maintain current species” and “help new species establish” were selected as adaptation goals at 43 % of sites; both “maintain current species” and “let ecosystem change” at 29 % of sites, and both “maintain current ecosystem and “let ecosystem change” at 17 % of sites.

There appeared to be various reasons behind selecting mixed goals in this way. In some cases, it appeared to be a “bet-hedging” strategy that balanced a desire to do everything possible to maintain current wildlife with a need to manage change. At several of the bigger sites, different goals and management regimes had been set for different parts of the site; for example, allowing change in one area while maintaining current vegetation and water conditions in another. This was sometimes the case when part of the site had been judged impossible to protect from the sea while another appeared feasible to maintain.

Similarly, the presence of a legal conservation designation in one part of but not all of a site had apparently sometimes led to different approaches in different places. Designated sites have specific features for which they are designated, and these features must be maintained in “favorable condition.” In several of the larger sites in which there were both designated and nondesignated areas, there was often a marked difference in the management approach being taken, with previous management being maintained in designated areas but with a relatively non-interventionist approach and less-specific or no targets for particular species the preferred option in nondesignated areas.

#### Planning Ecological Networks

At 29 sites (83 % of the total), planning decisions were taking into account the potential role of the site as part of an ecological network to support adaptation by species. This was most commonly being done at the scale of the site and its immediate environs, or within that region of England, but at a small number of sites, there was also consideration of networks at the national or the international level (generally as part of the network of European designated sites, Natura 2000) (Table 2).

**Table 2:** The number of sites considering ecological networks at different scales to support adaptation

Scale of ecological network considered in site management	Number of sites
Any	29
Creating networks within the site, or with immediate neighboring areas	17
Managing the site as part of a regional network	22
Managing the site as part of a UK-wide or international network	4

Data are shown for all 35 sites

### Management Actions

**Fig. 5:**
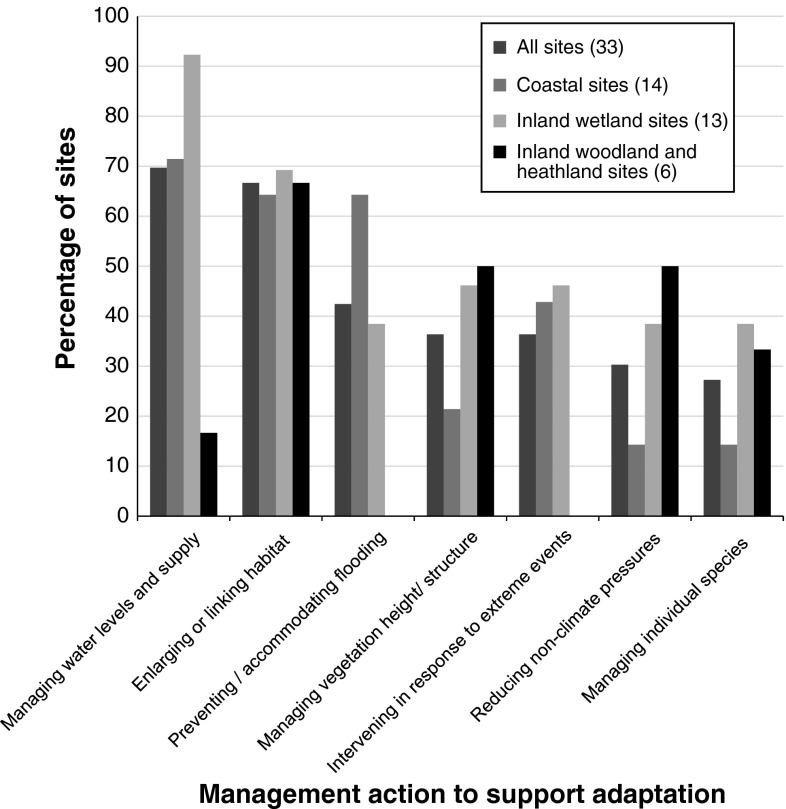
The percentage of sites at which different broad categories of management action were being undertaken to facilitate adaptation. These were chosen by respondents from a predetermined list in the questionnaire. Respondents could choose as many as they wished for each site. Data are shown—from *left to right*—for all the 33 sites at which adaptation was being considered, for coastal sites, inland wetland sites, and woodland/heathland sites

**Fig. 6:**
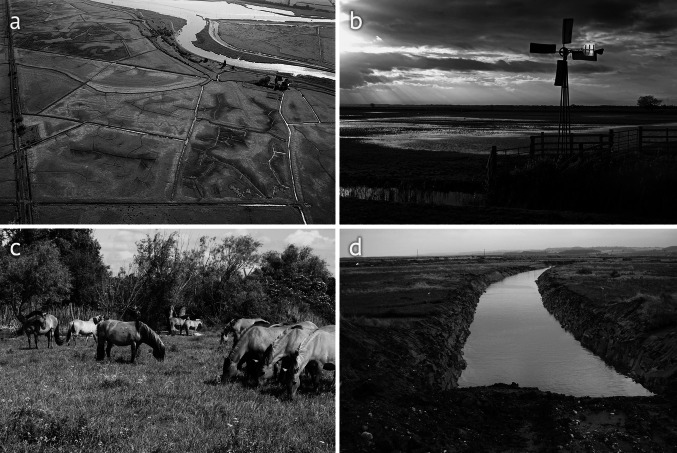
Some of the management work being carried out with adaptation to climate change in mind. **a** Aerial view of Berney Marshes in the Norfolk Broads. This freshwater wetland site is divided into three hydrological units that can be managed separately. A series of ditches and dams are used to keep water on site (www.mikepage.com); **b** wind pumps used for moving water between hydrological units at Berney Marshes (Kevin Simmonds); **c** Konik ponies grazing in new conservation land created by the Wicken Fen Vision project, the aim of which is to restore natural processes over a large area of former arable land in the Cambridgeshire Fens (Stuart Warrington, National Trust); **d** construction of a new river channel at Blakeney in Norfolk, to improve drainage of flood water from freshwater wetlands near the coast (Stuart Warrington, National Trust)

At eight sites, “minor” changes to management had been made in response to climate change; at 12 sites, “major” changes to management were reported (Table 3). Of the adaptation-specific management being carried out across the different sites, managing water levels and expanding suitable habitat for species were the two actions most commonly selected in the questionnaire (Fig. 5). The other management actions—manipulation of vegetation height and structure, prevention or accommodation of flooding, intervening in response to extreme events, acting to reduce nonclimate pressures to increase overall resilience, and management of individual species—were also reported at many sites. Not surprisingly, measures relating to water levels and flood management were much more common at coastal and inland wetland sites than at woodland and heathland sites. In some cases, management for adaptation was reported to be coordinated with other sites. For example, at one inland wetland site, abstraction of water was timed to minimize negative impacts on another site in the same river system. The following paragraphs give examples of the different types of management; some of these are illustrated in Fig. 6.

**Table 3:** Level of changes to site management made in response to climate change

Changes to management in response to climate change	Number of sites
Adaptation not currently being considered	2
Adaptation built into management at the outset	5
No real changes to previous management in response to adaptation goals	8
Minor changes to previous management to address adaptation goals	8
Major changes to previous management to address adaptation goals	12

(Respondents were asked to select one option for each site; they were free to interpret “minor” and “major” as they wished.) Data are shown for all 35 sites

#### Managing Water Levels and Water Supply

There were a range of aspects of management to manage water levels and supply. A large number of freshwater sites had put in place structures such as dykes, sluices, and pumps to store water during drought, to keep it out or to expel it during flooding, and/or to move it around the site. A few wetland sites had been constructed as a series of separate hydrological units with connecting pumps and sluices, giving managers the option of moving water between areas or holding it in a particular part of the site. During particularly dry years, water could be moved between the hydrological units through use of pumps and sluices to prioritize certain areas of the site and maintain them in good condition, even if other parts of the site had to be allowed to dry out.

#### Managing Habitat Patches and Networks

As noted above, many sites were considering ecological networks as part of setting conservation objectives. Management toward this goal often involved work to enlarge and connect habitat areas within sites. At a number of sites, it also included efforts to actively extend conservation management into surrounding farmland and increase species numbers outside formal reserve boundaries. In many cases this was being done through working with nearby landowners and helping them enter into agreements under the “Higher Level Stewardship” agri-environment scheme, which provides payments to land managers for environmental work. Several sites included in the study were formal large-scale conservation initiatives whose aims included coordinating management between, buffering, extending and in some cases eventually physically linking existing nature reserves. The strategies for achieving this include both trying to acquire land between existing reserves (and so eventually to create a very large single reserve) and working with private land owners to encourage more wildlife-friendly management on land outside formal reserves.

Another aspect of large-scale habitat management for adaptation was to create new areas of habitat to supplement and perhaps in time compensate for areas that could be lost or are at risk of becoming permanently unsuitable for current species. This approach has been taken particularly to create new inland wetland sites to address risks to coastal wetlands—several of the inland sites we surveyed had in fact been established largely to supplement some of the threatened coastal sites. The new inland wetlands included sites where reedbed and grazing marsh had been created on former farmland or former quarry sites. Besides freshwater habitat creation, there are a number of coastal sites in East Anglia where inter-tidal habitat is being created through managed realignment of coastlines to off-set losses at other sites. The most ambitious of these is the Wallasea Island Wild Coast project on the Essex coast, the largest coastal habitat recreation project in Europe. Here, a carefully designed network of new islands and intertidal areas will help compensate for areas that are being lost through sea level rise and coastal squeeze.

#### Managing Flooding

Various management actions were being taken to avoid or recover from flooding. This was particularly evident at coastal sites. Two coastal sites, one in Norfolk and one in Suffolk, had done major work to enable better drainage of water after storm surges. At both these sites, shingle ridges separate important areas of freshwater wetland from the sea. Previously, these ridges had been built up regularly to provide a defense against coastal flooding, but this left them vulnerable to being breached, following which the sites would be deeply flooded with salt water that took a long time to drain. In recent years, the approach had been changed to allow coastal processes to reshape the ridges into lower, wider structures. The wider profile of the ridge dissipated wave energy more effectively, and is less vulnerable to being breached, reducing the risk of prolonged saltwater flooding and consequent damage to the freshwater areas. The disadvantage was that the ridge was now more likely to have small amounts of seawater spill over the top during storm surges. Action had therefore been taken to improve the ability to drain and pump water out of the sites following flooding (including doing work to change the position of river channels that drain the sites).

At a third coastal site in Norfolk, changes in long shore drift processes and coastal erosion had been threatening the future of the reserve as a freshwater habitat. To address this, the shoreline had been realigned, causing the development of saltmarsh and brackish marsh which was expected to protect the freshwater further inland from tidal effects and allow freshwater habitats to be maintained. At a fourth site, on the Suffolk coast, banks had been constructed at the back of the freshwater reedbed to provide a refuge for fish during tidal flooding events; from this area, fish populations could reestablish after the saline water had retreated from the rest of the reedbed, allowing a more rapid recovery of the bittern (*Botaurus stellaris*) population that depended on the fish.

At many wetland sites, measures had been taken to improve the ability to pump floodwater out of the site. At one inland wetland that was subject to serious river flooding, new areas of freshwater habitat had been created on the site to replace areas that were often submerged during flooding.

#### Managing Vegetation

Management of vegetation included general work (usually as part of existing conservation management) to improve or maintain condition to increase overall resilience. In some cases, there had been modifications to these existing management practices in response to variable conditions. This included cutting reeds earlier in the year in response to earlier plant growth during mild winters, and delaying summer grazing in response to changes in the timing of bird nesting. At one site there had been a change toward more dynamic grassland management in response to more variable rainfall. In dry years, the grass was mown but in wetter years the vegetation was allowed to grow. This was reported to increase the resilience of the grass to drier conditions and benefit bird populations.

One adaptation-specific aspect reported for several sites (relating to hydrology as well as vegetation) was to manage sites for variation and heterogeneity in habitat structure (e.g., a range of water levels, vegetation of different types and at different stages of succession). Managers of larger sites appeared more confident of being able to do this, because of having a greater variety of soil, topographic and hydrological conditions and greater capacity to let natural processes act. In smaller, more isolated sites more intensive management was required to achieve heterogeneity, as natural processes were less strong (or might even, through succession of vegetation, be acting to reduce structural and habitat diversity). Highly artificial sites (of which there are many in East Anglia) were in a similar situation because the conditions that are currently valued are a direct product of human management and so required continued management to be maintained.

#### Responding to Extreme Events

Management responses to extreme events typically involved water management—either pumping water in during periods of drought or taking measures to pump or drain floodwater out, as outlined in some of the examples above.

#### Reducing Nonclimate Pressures

Examples given in respect of work to reduce other pressures focused particularly on measures to improve water quality, for example, by reducing pollution and removing sediment.

#### Managing Individual Species

Adaptation-related management of individual species included control of invasive species, for example, removing rhododendron or cutting back holly or bracken, and targeted management actions to improve habitat for priority mammal, bird or invertebrate species.

#### Experimental Management

At 16 sites (46 %), interviewees indicated that some management work had been carried out in an explicitly experimental way to test the effectiveness of new approaches. The experimental management work mentioned to us included testing the effects of different grazing regimes (both species of grazer and amount of grazing), of different approaches to managing vegetation, and of different protocols (timing and volume) for flushing saline water out of freshwater systems to achieve an appropriate balance between the need to remove salt water and the risk of introducing freshwater with too high a nutrient load from surrounding farmland.

Management work (whether experimental or not) was in many cases underpinned by various monitoring and surveillance activities (e.g., transect surveys of vegetation, annual surveys of breeding birds, dip-wells to measure water levels, and sampling to measure water quality). This helped managers to keep track of environmental changes, including possible impacts of climate change. For example, at one coastal site where the explicit aim was to research the functioning of natural processes and the impacts of climate change, the rate of saltmarsh accretion in relation to sea level rise was being monitored. At two of the large restoration areas, a joint program had been established with a nearby university to monitor vegetation, hydrology, and a range of species. As the sites developed, information was being collected on the dispersal of species into the new conservation areas and the formation of new species’ assemblages (Hughes et al. 2011).

Several interviewees also told us how monitoring had informed management plans in response to climate change. At one inland wetland site, the results of hydrological monitoring (including rainfall, water levels, and transpiration) were used to refine the water balance model for the site, which in turn informed the management plan. Monitoring of past change and good scientific studies had enabled some sites to identify when decisive action needed to be taken. At one coastal site in particular, analysis of historical trends in coastal processes had identified that erosion problems on the site were being caused by the confluence of two long shore drift processes. As a result of the analysis, managed realignment of the coastline was identified as the most appropriate option for extending the life of the freshwater reedbed at the site.

### Barriers to Action

**Fig. 7:**
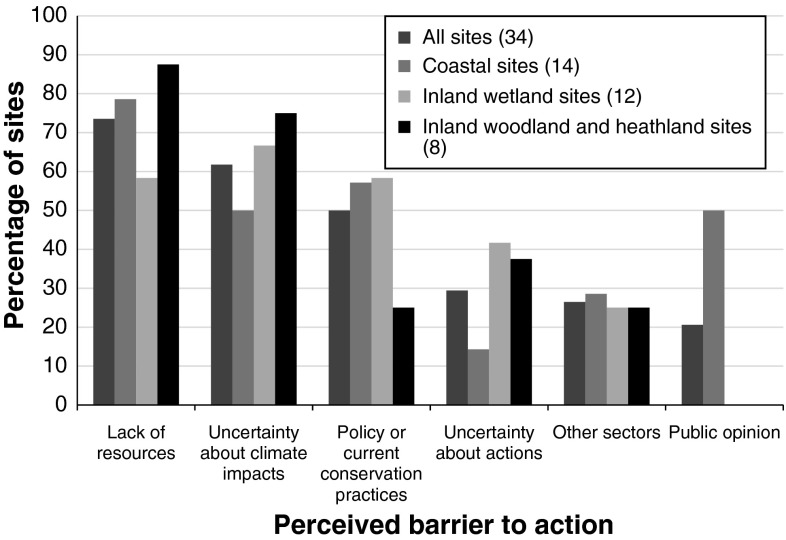
The percentage of sites at which different barriers to action were cited as a problem. The barriers listed were chosen by respondents from a predetermined list in the written questionnaire. Government policy and conservation practices and strategies were listed as separate categories on the questionnaire form, but are combined here as there was a high level of overlap in the issues respondents raised under these two categories. Respondents were asked to list the three greatest barriers to action at each site, though in a few cases more than three were mentioned as important and have been included in the data presented here. Data are shown for—from *left to right*—all sites in the study, excluding one for which information was missing; coastal sites; inland wetland sites; and woodland and heathland sites. The two sites at which adaptation was not being considered are included here as the site managers were still able to identify barriers to adaptation in the future

“Lack of resources” and “uncertainty about the impacts of climate change” were the most commonly cited barriers to taking effective adaptation action (Fig. 7). Current natural resource management policies or conservation strategies were also mentioned as a potential barrier at many sites. Uncertainty about appropriate action to take was mentioned infrequently relative to uncertainty about potential impacts. A small number of sites cited the influence of other sectors (particularly in relation to water use/water quality). There were differences in some responses among different types of sites. In particular, there was an apparent difference between coastal and inland sites: at coastal sites, there appeared to be less uncertainty about how to respond to climate change, but 50 % of coastal sites mentioned public opinion as a potential major barrier to action. (In contrast, no inland wetland, woodland, or heathland sites mentioned public opinion as a major barrier in questionnaire responses, though it was mentioned as a potential minor issue in interview discussions). The following examples illustrate some of the potential constraints faced at different sites:

#### Resources

Land, staff time, and expertise were often mentioned as limiting resources. Land prices were mentioned a number of times during interviews: much of the land in East Anglia is high-quality agricultural land and prices were high, limiting options to expand existing sites or create new sites. In some cases, a perceived lack of monitoring and information about environmental change at the site level was mentioned as a potential barrier to taking action.

#### Conservation and Natural Resource-Management Policies

Water management was raised many times as an important issue, with constraints reported in some cases in relation to other sectors (see below). Some site managers suggested that the requirement to maintain features for which sites were designated was a potential constraint on adopting more flexible or innovative management, though there were differing opinions among the people we interviewed as to whether current conservation designations are a real constraint to changing management decisions.

#### Interactions with Farming

Maintenance of currently valued habitats and landscapes at several heavily modified sites relied on grazing by large herds of cattle. Livestock were provided by local farmers, but if climate change affects the profitability of grazing in the area (which was already much reduced from earlier levels) and animals were no longer available, then it could have an impact on conservation and potentially make current management unfeasible. At a separate set of sites, it was reported that reserve managers would like to be able to store water in winter to increase resilience to drier summers, but that this was constrained to some extent by the area being drained throughout the year for arable farming.

#### Coastal Defense

Sea defenses designed to protect settlements on the Essex coast from tidal flooding had altered patterns of erosion and long shore drift, preventing sediment accreting at one coastal conservation site. These changes in coastal processes, coupled with sea level rise, meant that the site was increasingly vulnerable to tidal flooding.

#### Views of Local Landowners and Communities

Public attitude toward issues such as managed realignment of coastlines and food security might also constrain future adaptation. As noted above, managers of many coastal sites in particular mentioned that public opinion was an important factor to consider in the management of coastal flooding, as there could be resistance to managed realignment and abandonment of sea defenses if people felt that they would no longer be protected from coastal flooding. At one inland wetland site, concern over food security and opposition by land owners was suggested as a possible barrier to further expansion of conservation land, and the site manager also noted that there might be resistance to changing management practices if this resulted in changes to valued cultural landscapes.

### Sources of Information Used to Inform Adaptation

Experience of past weather events and environmental conditions was reported to be informing adaptation management “a bit” at 12 sites, “a lot” at 11 sites and “almost entirely” at three sites. Many of the practical actions reported to us as management for adaptation had been implemented in response to past events. These included

- Measures (including dykes, sluices and pumps) to store water during drought, to keep it out or to expel it during flooding, and/or to move it around the site in response to recent droughts and floods.
- Compensatory habitat areas to replace areas that had been flooded in the past and were thought to be at risk of future flooding.
- Sluices to enable saline water to be flushed out of freshwater areas.
- Establishment of fire breaks following a recent fire.

**Fig. 8:**
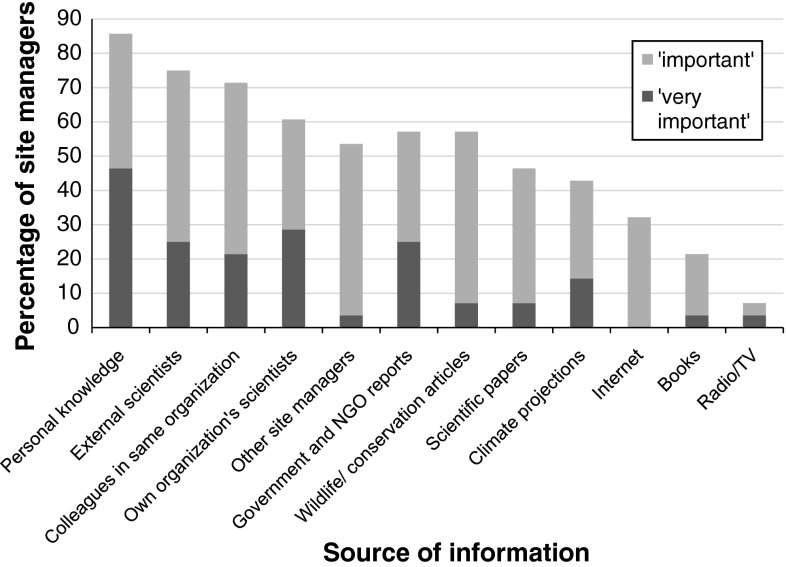
The percentage of the 28 survey respondents (“site managers”) that cited each source of information (from the list given in the questionnaire) as important or very important in informing adaptation at their site(s). The five categories on the left of the graph relate to direct advice from other people; those on the right to written publications. Respondents were able to select, from a set list, as many options as they wished for each site. Where the same person provided multiple questionnaire responses for separate sites, information has been combined, with each information source mentioned by that person being counted only once

Respondents were asked to indicate which sources of information—from a list provided in the questionnaire—they found “important” or “very important” in informing responses to climate change. The five response options relating to gaining information through personal contacts were selected more often than those relating to using published information. In particular, use of personal knowledge, scientists, and other colleagues in a site manager’s own organization, and external scientific advisers were selected most often, and were most often indicated to be “very important” as sources of information. Among the different types of published information, reports, and articles on wildlife and conservation in publications such as *British Wildlife* were most commonly mentioned as being important or very important (Fig. 8).

## Discussion

### Key Themes

The primary goal of this study was to explore how examples of existing adaptation in conservation areas might help to bridge the gap between principles and practical action and provide lessons for supporting future adaptation. Six important themes were apparent across many sites. Each of these themes is discussed below, highlighting distinct aspects of each theme, including different strategies taken by different conservation sites, and trying to draw out lessons that might apply for adaptation in general.

#### Building on Past Experience

Many of the site managers we spoke to were already concerned about, and were responding to, current and near-term variable and extreme weather/environmental conditions. In some cases, adaptation to climate change had been part of their thinking for quite a few years. Many site managers had been able to use past experience of weather events (including changes in rainfall, drought, warmer temperatures, and floods) to inform their planning and management for future climate change.

This suggests that adaptation, in a general sense, is not an entirely new issue for conservation. To a certain extent, management of nature reserves and protected areas has always been about managing change. Climate change appears likely to increase the speed and magnitude of change, not necessarily in predictable ways. However, past experience and knowledge of a particular site, and existing good conservation practice to reduce vulnerability to current pressures, often appear to provide a solid foundation for future action in the face of uncertainty.

#### Resisting or Accepting Change

At many of the sites surveyed, it was apparent that there was a strong aspiration to try to conserve current species and ecosystems, at least for the foreseeable future. However, there was often an acknowledgment of also needing to accommodate change. The majority of interviewees indicated that change would be inevitable at some point, at least in the longer term. In the short to medium term, a range of strategies relating to resisting or accepting change were evident.

*Maintaining current conditions* appeared to be most appropriate under two sets of circumstances: (1) sites where there are major potential impacts on the horizon (particularly sea level rise and associated flooding, or shortages of freshwater), but high confidence that the events would not occur for several decades. This was particularly the case when the sites were well-established and had a valued set of species that might provide source populations for other nature reserves. (2) Sites at which expected changes appear to be quite subtle or there was uncertainty about which of two or more opposing potential changes would prevail.

*Accepting change* appeared to be most appropriate: (1) when there are thought to be major impending impacts and little chance of reducing exposure to them (in our study, this was found particularly at some coastal sites); (2) where dynamic natural processes are fundamental to the functioning of the site and the focus of conservation objectives; (3) where change presents an opportunity, for example, to acquire new species, or to expand a particular ecosystem type, or simply to observe how species assemblages and ecosystems change in response to changing conditions. In addition, in newer conservation areas that are not legally designated, there is potentially more flexibility to change management to take into account of climate change and use more novel management techniques.

As our results show, different strategies could be more appropriate at different sites. In addition, often a mix of goals and strategies can be adopted within a single site, if pressures, constraints and opportunities differ in different areas within the site boundaries.

#### Coping with Variable Conditions

Variability (as opposed to permanent directional change) emerged from the interviews as one of the climate-related issues that appeared to be uppermost on site managers’ minds. Managers of many reserves appeared to be modifying their goals and targets to accommodate temporal and spatial variabilities. Several interviewees even noted benefits of variability. It was suggested that different conditions at different times would benefit different species, which might even enhance meta-population processes and overall biodiversity (provided that there was a functionally connected network of areas in which there were some suitable habitats at all times). Our study shows the wide range of strategies that exist to deal with or even take advantage of different aspects of variable conditions:

*Changing the timing of existing management actions* in response to variable conditions.

*Setting flexible targets* that aim for average species numbers or environmental conditions (such as water levels) over several years rather than annually. Under some circumstances, this could be extended to taking an even more open-ended approach that focuses on natural processes with few or no targets for individual species.

*Prioritizing core areas* by focusing resources to keep some parts of a site in good condition for valued species even if other parts have to be allowed to become temporarily unfavorable. The best examples of this in our study were wetland sites with separate hydrological cells, where water could be preferentially pumped into one cell during drought. This approach of prioritizing the most important areas during periods of variability could in theory be extended to groups of nearby sites, ensuring that at least one site was maintained in good condition even if others could not be.

*Accepting variability in order to reduce the risk of catastrophic events* As noted in the results section, two coastal sites had done work on their sea defenses that reduced the risk of breaches and major flooding but at the cost of increasing the frequency of small amounts of seawater spilling over the top. This was, in effect, a conscious decision to accept more variable conditions (in the form of more frequent minor floods) in order to reduce the risk of major events.

*Increasing heterogeneity and creating habitat mosaics* As noted above, one approach commonly reported as a response to variable conditions was to manage sites for variation and heterogeneity in habitat structure. The rationale for this was that it would maximize the chances that suitable niches for species would be available somewhere on the site even in generally unfavorable conditions (e.g., cooler wetter areas for species to shelter in during drought). In this way, increasing heterogeneity can be thought of as a form of risk hedging (Stafford Smith et al. 2011).

#### Large-Scale Approaches and Networks of Sites

As in many other countries, the concept of ecological networks (Jongman 1995; Jongman et al. 2004; Lawton et al. 2010) is prominent in current conservation policy in England (Defra 2011a, b). Large-scale approaches to conservation (both larger sites and better management coordination and functional links between them) have been suggested as an important response to a future in which landscapes and the ecosystems and species populations they support appear likely to become increasingly dynamic (Opdam and Wascher 2004; Hopkins et al. 2007; Vos et al. 2008; Heller and Zavaleta 2009). In East Anglia, climate change appeared to be leading conservation managers to consider how their individual sites fit into the wider landscape and network of other sites around them, and, in some cases, toward an increased focus on a strategic, large-scale approach to adaptation. The work they were doing shows some of the different ways in which “landscape-scale” conservation can be approached, at various scale and levels of complexity:

*Coordinated management among sites* There appeared to be a high level of informal cooperation and coordination between some groups of nearby sites managed by different organizations, particularly on the north Norfolk coast and Suffolk coast and in the Norfolk Broads. While conservation objectives and the approach to management were decided at a site level, there was interaction between the sites, recognizing the ecological processes (particularly dynamic coastal processes) that link them. Such coordination presumably would inform future decisions about resisting or accepting either permanent or temporary change at any one site.

*Extending conservation management beyond reserve boundaries* through land acquisition or working with private landowners. This can create habitat to support new species populations that could survive outside a nature reserve and potentially recolonize it if conditions became temporarily unsuitable, as well as to help in buffering the core site from external pressures such as diffuse pollution.

*New sites to provide supplementary and compensatory habitat* As noted in the results, some entire new inland wetland sites have been created in East Anglia to address risks to coastal freshwater wetlands (Sills and Hirons 2011). The primary reason has been to create new habitat for bitterns; the majority of the small UK population of this species is confined to coastal freshwater wetlands in East Anglia and is vulnerable to coastal flooding (Gilbert et al. 2010; Ausden 2013), but it is hoped that the new areas will support a range of other wetland species, including potential new arrivals. There are also several coastal sites where inter-tidal habitat is being created through managed realignment to off-set that is being lost at other sites (Ausden 2013).

*Large*-*scale ecological restoration and re*-*creation* Several sites surveyed were, or were part of, ambitious large-scale restoration projects that aim to create better ecological connections between existing sites and greatly enlarge areas under conservation management. The people leading the work hoped that it would not only increase the total area of habitat available for species but also create greater potential for natural processes to occur, species to move between areas, and a range of niches to be naturally provided; all of which should support adaptation to climate change (Boyd et al. 2008; Lawton et al. 2010).

#### The Role of Other Sectors

East Anglia is an intensively managed area, and in fact many of its conservation sites have to a large extent been shaped by historical human management. Therefore, it was not surprising that other sectors were mentioned frequently as being involved in both problems and solutions in relation to adaptation. The general issue, however, is likely to be relevant to conservation sites in many other places. As our study shows, it can present both constraints and opportunities.

*Constraints* The achievement of both existing conservation goals and future adaptation at many sites was reported to be constrained by factors such as livestock farming, drainage for arable farming, abstraction for public water supply, and flood defenses designed to protect urban areas, as summarized in the examples in the results section above. (At least, one site manager said that existing nonclimate pressures, from development and atmospheric pollution, overshadowed and took precedence over dealing with longer-term change.) In addition, in some cases, climate change was thought likely to exacerbate some of the existing problems caused by the activities of other sectors.

*Opportunities* It is important to note, however, that the actions of other sectors were not always seen to be a constraint to adaptation. There were examples of solutions that would benefit both conservation and other sectors. For example, at one wetland site, changes to water management regimes had not only aided adaptation but also benefitted farmers in agri-environment agreements who were trying to create grazing marsh. At one inland wetland site, new approaches to water management and drainage were being discussed, with a potential opportunity for conservation organizations to work with the Internal Drainage Board to store water on the site. A number of opportunities for delivery of multiple benefits and ecosystem services had been identified. For example, several sites were providing, or had the potential to provide, flood protection for nearby human settlements. There were also examples of conservation organizations working with other sectors, including infrastructure development companies and gravel extraction companies, to create new conservation sites that would deliver adaptation benefits.

#### Adaptive Management

The concept of adaptive management (Holling 1978; Walters and Holling 1990) is often promoted as a core part of adaptation, as it is a flexible approach that explicitly addresses uncertainty. Applied properly it is an active approach involving “learning by doing” (Walters and Holling 1990), “gaining knowledge and using it to modify practices to achieve management goals” (Lindenmayer and Burgman 2005); it includes not just careful monitoring and documentation of management applied, but also the development and testing of hypotheses about the system and how best to manage it, and the modification of management actions according to the results. Good examples of a complete adaptive management approach being applied in practice are, however, somewhat rare.

Collectively, the sites surveyed in this study appear to provide some good examples of at least some of the components of an adaptive management approach. Many interviewees mentioned the importance of taking a step-by-step approach, basing management on past experience and avoiding taking steps that would be hard to reverse. There was, in addition, a strong apparent awareness of the need to monitor changes and the effects of actions, and in some cases, of the role of testing new approaches.

The importance of monitoring was clear. At many sites, monitoring was thought to be important not just for identifying the impacts of climate change but also for reviewing management plans in response to climate change. Monitoring underpins (or should underpin) many of the adaptation strategies we have highlighted above. As noted in the results, we also found some examples of experimental approaches being taken, ranging from small to very large-scale experiments. This included trying out a range of new techniques and trying to learn from the results. In some cases, this was to prepare for a likely future threat, in others to learn about what different management approaches could achieve, to be as prepared as possible for changes in species assemblages that might occur in future.

### Does Adaptation on the Ground Reflect Theory and Principles?

Many of the goals and management actions we recorded are in line with various principles for adaptation and for conservation in general, particularly those published specifically for England. These include the need to conserve existing biodiversity as a starting point for adaptation, accommodating change, increasing connectivity, maintaining varied habitat structure and water conditions, reducing nonclimate pressures, and integrating climate change into planning exercises (Hopkins et al. 2007; Smithers et al. 2008; Heller and Zavaleta 2009; Lawton et al. 2010).

A range of different typologies have been developed to categorize adaptation measures. A distinction has been made in the literature between actions that “build adaptive capacity” and those that “deliver adaptation” (UKCIP 2010); between “autonomous” and “planned” adaptation (Fankhauser et al. 1999; Smith et al. 2000; Füssel 2007); between “reactive” and “anticipatory” adaptation (Parry et al. 2007); and between “incremental” (or resilience-focused) and “transformative” or “transformational” adaptation (Stafford Smith et al. 2011; Kates et al. 2012; Morecroft et al. 2012; Palutikof et al. 2013). The approaches and actions being taken by conservation managers in our study do appear to fall along a spectrum that can be related roughly to some of the typologies above. At various points along this spectrum we can identify examples of:

- sites carrying out environmental surveillance and perhaps testing new approaches (and so building knowledge and capacity) without any changes to overall management.
- sites making small incremental changes, involving modifications to existing conservation targets and management operations. (Such examples could in a sense also be seen as “autonomous” and reactive as they are often changes to existing management and in response to recent or current weather conditions.)
- sites that had kept their existing conservation objectives but were carrying out major new work to maintain the site in its current condition.
- sites accepting the need for a greater level of flexibility in their existing targets.
- sites at which there had been major changes to conservation goals, particularly acceptance or facilitation of changes such as freshwater reedbeds becoming saline wetlands, and associated major management actions such as engineering works of coastlines or watercourses, and creation of whole new sites to replace existing ones under long-term threat (the majority of these examples are certainly “planned” and “anticipatory,” as well as “transformative”).

The incremental/transformative adaptation typology is of great relevance to conservation, and is the subject of growing discussion among conservation scientists and practitioners. It relates to a fundamental question: Does adaptation mean the continuation of current conservation measures with only minor adjustments or does it necessitate large changes? As with many questions of this sort in conservation, the answer will vary from place to place. But the information we have collected in this study, and the themes outlined above, help us identify some of the factors that might influence the decision. The transition from “resilience” through “accommodation” to “transformation” can be considered both for different elements of ecosystems and at a range of spatial scales (Morecroft et al. 2012). Some of our examples (and potential future scenarios for these sites) help in illustrating this.

Different elements of an ecosystem—individual species, communities, overall ecosystem structure, and function—will not always be affected by climate change at the same rate. Thus, the overall ecosystem at a site could remain resilient even if many of the species it supports are no longer present and are replaced by new species. This potentially could occur at some of the sites in East Anglia (e.g., some inland wetlands) at which there might be reasonable certainty of maintaining current ecosystem or vegetation types, but changes in other factors might over the course of time cause changes in species assemblages. Under these circumstances, it might be appropriate to take a “resilience” approach to the overall system but one of “accommodation” in relation to changes to individual species. In other cases, transformation of the whole system and its components could occur if some major tipping point is reached; the obvious example being coastal freshwater wetlands changing to brackish or saline systems, something that some of the sites in this study were already facing.

Major transformation at one scale might not affect, or in fact could even aid, resilience at a larger scale. This is evident even within some of the sites we studied; a number of coastal sites had allowed or even facilitated coastal realignment and the incursion of salt water into freshwater areas in order to improve protection of the remaining part of the site. Wetland sites prioritizing some hydrological cells over others in drought years could be seen as an analogous approach—accepting major (albeit temporary) change to some areas allows resources to be focused to maintain core areas and so promote the resilience of the bird and other species populations in the site overall. At a larger scale, transformation has already occurred in some places—coastal realignment at some sites, entire new inland wetlands created—and it appears likely that in the longer term, further coastal changes are inevitable; however, at the scale of East Anglia as a whole, the overall desired effect is to maintain the resilience of the freshwater wetland network and the valued species, such as bittern, it supports.

This illustrates how taking a large-scale view could help conservation planners to consider the resilience of an overall site network and to identify where new sites might need to be created to supplement and replace threatened existing ones. A strategic overview should also help in informing the setting of appropriate targets for each site, based on where current species are or are not expected to persist and where new species might colonize (Hole et al. 2011). Recent research into bird populations in Special Protection Areas in the UK suggests that resilience of populations to climate change will be maximized if sites are managed as networks “with considerable capacity for turnover between sites”(Johnston et al. 2013). Linked to the spatial aspect, there is also a temporal consideration. Not all climate impacts will occur at the same time in every place. From discussions with site managers there was clearly a strong desire to maintain some high-quality but threatened sites in their current condition in the short to medium term, to preserve source populations of species and buy time for the creation of new sites.

### Implications for Supporting Practical Adaptation

We believe that there is great scope for the lessons being learned on individual sites to be shared among sites, and among the different conservation organizations that manage them. Collective experiential knowledge has an important role to play in conservation (Fazey et al. 2006), and our results highlight how important direct advice and information is for conservation practitioners. Several people we interviewed said they would like more regular opportunities for reserve managers to discuss their experiences, management approaches, and the results of new techniques. There is probably also scope to improve the recording and collation of the results of conservation interventions, particularly the more experimental approaches, for example through the conservationevidence.com site. This would enable lessons to be shared and the outcomes of different approaches to be properly evaluated (Sutherland et al. 2004). Even within this small study, we found a range of approaches being taken to similar problems at different sites; there is value in a diverse range of approaches (Millar et al. 2007; Lindenmayer and Hunter 2010) and great potential to learn from the results, if appropriate monitoring and evaluation are done. There is also a clear need for new scientific information to be communicated effectively to site managers, who often will not have time for extensive reading of the scientific literature.

One of the challenges of adaptation, especially when considering the management of complex natural systems, is uncertainty about the type, timing, and magnitude of future changes. Because adaptation is so place-specific, it is challenging to provide precise, practical guidelines for conservation practitioners. Our findings highlight that adaptation is not simply about predicting a future directional change and preparing for that eventuality; preparing for unpredictable and variable conditions may be just as important. This provides a clear starting point for action as there should be experience of past extreme events to draw on. But it does not necessarily mean continuing with“business as usual.” It is clear that in some places, whether in the short or long term, seeking to maintain the resilience of current systems in their current states could becoming increasingly costly or untenable, presenting site managers with difficult decisions to make.

For both individual conservation sites and site networks, climate change appears to create a need to plan further ahead, something that is already being done by some conservation organizations (Ausden 2013). Adaptation decisions can have long lifetimes (Stafford Smith et al. 2011), and we have seen that, at some sites, adaptation planning is being considered over fairly long periods (in some cases, looking out to a horizon of twenty years or more). Ideally, planning should include consideration of the different adaptation “pathways” that could be taken at each particular site, the implication of choosing each pathway, and the future environmental or other changes that might require a decision to change from one path to another (Wise et al. 2014). To aid this, further research is required to overcome our relative lack of knowledge about ecological tipping points. A number of reports and papers have been published recently to provide adaptation guidance and tools for practitioners (e.g., Glick et al. 2011; Oliver et al. 2012). We suggest that the main themes and issues we have identified from this study could also make a contribution to adaptation planning by being developed into a series of questions for reserve managers to consider when planning adaptation at their site and considering possible paths to take.

## Conclusion

This study aimed to help in bridging the gap between principles and case studies for adaptation in nature conservation, and in providing practical examples of how conservation managers in the field are addressing adaptation. Our results, like those of other studies, show that there is no “one-size-fits-all” response to climate change; appropriate decisions will be informed by a range of factors specific to each site, and will probably be most effective when taking into consideration changes and management actions at other sites. Our findings also highlight that whether adaptation requires action that is significantly different from existing conservation management depends both on the factors affecting the site and on the spatial and temporal scales at which the question is viewed. The themes identified in this paper provide, we believe, not only an illustration of what adaptation in nature conservation can entail in practice but a framework of issues and questions that managers of conservation areas can consider when thinking about how to respond to climate change. Though the answers will be specific to a particular place and point in time, the general questions are likely to be relevant to most if not all conservation areas.

## Electronic supplementary material

Below is the link to the electronic supplementary material.

Supplementary material 1 (DOC 42 kb)
