# Supplementary material for: Adaptation in Practice: How Managers of Nature Conservation Areas in Eastern England are Responding to Climate Change
Source: Environ Manage. 2014 Mar 20;54(4):700–19. doi: 10.1007/s00267-014-0254-6 (PMC4171586; doi:10.1007/s00267-014-0254-6)
Supplement: Supplementary file 1 — Supplementary material 1 (DOC 42 kb) [file 267_2014_254_MOESM1_ESM.doc]

**Supplementary material for the manuscript ‘Adaptation to climate change in protected areas: lessons from conservation action in England’**

**Summary of the written questionnaire used in this study**

The questionnaire was on a spreadsheet, with questions requesting one or more options to be selected using drop-down menus. Space was also provided for free text responses to all questions, and respondents were invited to provide notes to elaborate on the categorical answers given. The completed questionnaire was later used as the basis for the semi-structured interview.

***Part 1. Background information***

**Q1. What is the name of the site?**

**Q2. Which National Character Area is the site in?**

**Q3. Please describe your general role in the site (e.g. reserve manager).**

**Q4. Which of the following broad vegetation/land cover types occurs in the site?**

[Respondents invited to select any that account for a significant part of the site and its conservation interest, from a list of UK Biodiversity Action Plan habitat types]

**Q5. Approximate size of the site in ha**

**Q6. Which of the following statements best describe the land ownership and management arrangements on the site?**

[Respondents invited to select as many options as relevant from a list of different possible land tenure and management arrangements]

**Q7. What are the primary conservation goals of the site?**

[Respondents invited to select from a list of options]

***Part 2. Impacts of climate change***

**Q8. Has an assessment been made of how the natural environment in your site might be vulnerable?**

[Respondents asked to select one option from:

No, vulnerability has not been assessed in any detail;

Yes, a simple vulnerability assessment has been done, based on general ecological knowledge and general published information and/or using results of studies in other areas;

Yes, a detailed vulnerability assessment has been done for this specific reserve]

**Q9. If a vulnerability assessment has been undertaken, was it done in collaboration with other conservation sites in the area?**

**Q10. What possible consequences of climate change do you think are likely to have the greatest impact on the species and ecosystems on the site?**

[Respondents asked to select as many consequences as applied, from the following list:

Effects of changing species distributions as a result of changing temperature and rainfall patterns (e.g. valued species no longer being able to survive in their current ranges; new species becoming established; changing ecological communities)

Effects of changing seasonal events and longer growing seasons (e.g. changing plant growth, phenological mismatch)

Effects of drought + high temperatures (including fire)

Effects of river flooding

Effects of sea level rise and coastal flooding

Effects of extreme precipitation and storms

Effects of changes to human behaviour as a result of climate change (e.g. changed farming practices, water use, recreation)

Effects on water quality of changing rainfall patterns and rising temperatures

Effects on aquatic ecosystems of changes in stream flow]

**Q11. At what point in time do you think the impacts you noted above [in Q10] will become a serious issue for the achievement of your conservation goals and how the site should be managed?**

[For each answer to Q10, respondents asked to choose one of the following:

Now/in the next few years;

Within ten years;

Within 20 years;

Within 50 years; Longer than 50 years;

Unlikely ever to be a serious issue compared with other pressures]

***Part 3. Integrating adaptation into conservation goals***

**Q12. To what extent is adaptation to climate change currently a factor in the design, planning and management of the site? (Regardless of whether it has led to changes in previous management.)**

[Respondents asked to select one option from:

Not at all;

Adaptation is a minor factor in management plans/actions;

Adaptation is a major consideration in management plans/actions;

The conservation area was established with adaptation as a central objective from the outset]

**Q13. For how long has adaptation explicitly been part of the planning and management of the site?**

[Respondents asked to select one option from:

Less than one year;

One to two years;

Three to five years;

Six to ten years;

More than ten years]

**Q14. In broad terms, which of the statements below best describe your main goals in relation to adaptation?**

[Respondents asked to select an unlimited number of options from a list that included:

Maintaining existing populations of particular species or groups of species in spite of climate pressures;

Increasing ecological connectivity to enable species to move within/ through/ in and out of the area;

Enabling new species to become established in the conservation area;

Maintaining the overall ecosystem in its current structure/state;

Letting the ecosystem change, or actively helping it to do so (e.g. letting a freshwater wetland change to brackish/saline)]

**Q15. Have you set specific qualitative or quantitative targets to measure progress towards these goals?**

**Q16. Is the adaptation work being done on your site intended to make a specific contribution to enhancing ecological networks and connectivity (e.g. as part of wider work your organisation is doing)?**

**Q17. If you answered yes to Q16, over what sorts of spatial scales are the ecological networks to which your site contributes being considered/developed?**

[Respondents asked to select an unlimited number of the following options:

Increasing connectivity within the site itself;

Management of the site is being planned as part of a wider regional-scale ecological network;

Management of the site is being planned with reference to large-scale species movements across England, or across Europe (e.g. as a site to accommodate new species arriving from southern England or from the continent)]

**Q18. Are the ecological networks mentioned above being designed with the movement of a particular species or group of species in mind?**

**Q19. Have any particular tools or methods been used to determine the physical structure of these networks? (E.g. size and shape of core habitat areas, length and type of corridors, distance between patches)**

**Q20. Does your planning of ecological networks involve cooperation with other conservation sites in the area? (e.g. to consider species movement between sites, or consider how one site might provide habitat to replace habitat lost in another site)**

***Part 4. Management actions and monitoring***

**Q21. On the basis of the projected climate impacts and adaptation goals above, have your management actions changed?**

[Respondents asked to select one of the following:

previous management (i.e. what was being done before climate change become a consideration) was deemed adequate for adaptation, without changes;

previous management was deemed appropriate with some small changes (e.g. in timing/extent of actions);

Management actions have been significantly changed to address adaptation, and/or new management actions introduced.]

**Q22. What do you consider to be the most important specific actions that you are taking on your site to help the natural environment adapt?**

[respondents asked to select an unlimited number of the following options:

Species-level management (e.g. controlling invasive species, supplementary feeding; re-introduction)

Enlarging, buffering and linking habitat patches or creating new patches (including compensatory habitat to replace other areas)

Maintaining or altering the structure of vegetation (e.g. increasing heterogeneity of vegetation; changing vegetation height; planting trees for shade)

Managing water levels/water supply

Directly intervening in response to extreme events (e.g. fire fighting, pumping water after flooding, pumping water in during drought)

Measures to protect against or safely accommodate flooding

Actions to reduce non-climate pressures on the environment (e.g. water pollution)]

**Q23. Are any of these management actions coordinated with other nature reserves?**

**Q24. Do you monitor changes in any of the following on your site?**

Species (flora)

Species (fauna)

Habitats/vegetation types

Physical processes

Water quality

Recreational use

**Q25. Are any of your management actions being undertaken in an explicitly experimental way, with the results recorded, analysed and used to modify future management? (For example, are you testing and comparing different management approaches to determine which works best to achieve a particular goal?)**

***Part 5. Sources of information used; barriers to action***

**Q26. To what extent would you say your approach to managing the site to cope with future environmental change is based on your experience of past changes in the area, such as past extreme weather events?**

[Respondents asked to select one of:

Not at all

A bit

A lot

Almost entirely]

**Q27. What other sources of information have been most useful/important in helping you to understand possible climate impacts, incorporate adaptation into your conservation goals, and identify the necessary management actions?**

[Respondents invited to select an unlimited number of the following, and indicate whether ‘important’ or ‘very important’

Personal ecological knowledge and experience of project staff

Scientists in your own organisation

Other colleagues in your own organisation

External scientific researchers

Other conservation site managers in the region

Other experts

Climate projections (UKCP09) and/or modelled outputs of impacts (e.g. flow rate, water quality)

Reports published by government or NGO organisations (e.g. biodiversity adaptation principles)

Articles in scientific journals

Other journals or magazines (e.g. British Wildlife, ECOS, New Scientist)

Information from the internet

Books

Newspaper/radio/TV

Other published information]

**Q28. Which of the following, if any, do you feel are currently the greatest barriers to taking action to adapt to climate change on your conservation site?**

[Respondents asked to select the three most important factors]

Uncertainty about climate impacts and how they will affect complex ecosystem processes and species interactions

Lack of knowledge about appropriate actions to take in response

Lack of resources (e.g. land/money/staff)

Current conservation practices and strategies in England

Government policy

Public opinion

Difficulty influencing other sectors/taking necessary action outside the site]

**Q29. Do you think climate change creates any opportunities for conservation on this site?**
